# The Partners in Health scale for older adults: design and examination of its psychometric properties in a Dutch population of older adults

**DOI:** 10.1111/hex.12488

**Published:** 2016-10-07

**Authors:** Karin Veldman, Sijmen A. Reijneveld, Maarten M. H. Lahr, Ronald J. Uittenbroek, Klaske Wynia

**Affiliations:** ^1^ Department of Health Sciences, Community and Occupational Medicine University Medical Center Groningen University of Groningen Groningen The Netherlands; ^2^ Department of Neurology University Medical Center Groningen University of Groningen Groningen The Netherlands

**Keywords:** Partners in Health scale, psychometric properties, self‐management behaviour

## Abstract

**Background:**

Self‐management is an important asset in helping older adults remain independent and in control for as long as possible. There is no reliable and valid measurement instrument to evaluate self‐management behaviour of older adults.

**Objective:**

This study aims to design a measurement instrument, that is the Partners in Health scale for older adults (PIH‐OA), to assess self‐management knowledge and behaviour of community‐living older adults and to examine its psychometric properties in a Dutch context.

**Methods/design:**

The original PIH scale was translated into Dutch and adapted to the context of community‐living older adults, resulting in the PIH‐OA. Data for 1127 participants (mean age 81.7, SD=4.5) from the Embrace study were used to assess the psychometric properties.

**Results:**

Data fitted a three‐factor model, covering the constructs Knowledge, Management and Coping, with good internal consistencies (Cronbach's alphas ranging from .77 to .84). Known groups validity was confirmed: no differences were found between gender, age and marital status groups, and differences were found between the education level and health status groups. Discriminant validity was confirmed by weak correlations between PIH‐OA scales and scales evaluating “Perceived integrated care” and “Activities of daily living (ADL)” (*r*<.30), and a moderate correlation between the PIH‐OA subscale “Coping” and the scale evaluating “ADL” (*r*=.41).

**Conclusion:**

The PIH‐OA appears to be a reliable and valid measurement instrument for assessing the self‐management knowledge and behaviour of older adults. This could help professionals provide tailored support to improve the well‐being and independence of older adults.

## 1 Introduction

Self‐management is a key element to improve well‐being in older adults.^1^, ^2^ Maintaining self‐management is of utmost importance for all older adults as a precondition for remaining in control and independent for as long as possible.^3^ Furthermore, efficient self‐management can help older adults communicate effectively about their needs with their health‐care providers, which can lead to better provision of efficient and tailored health care.^4^ To set the level of self‐management of older adults, reliable and valid measurement instruments are important.

In the existing literature, self‐management is defined in different ways. Lorig^5^ stated that self‐management behaviour :enables participants to make informed choices, to adapt new perspectives and generic skills that can be applied to new problems as they arise, to practice new health behaviours and to maintain or regain emotional stability”. Battersby et al.^6^ and Petkov et al.^7^ defined a good self‐manager as someone who “(i) has knowledge about his or her condition, (ii) follows a care plan agreed with health‐care providers, (iii) is actively engaged in decision‐making with health‐care providers, (iv) manages and monitors symptoms and signals of his or her health condition, (v) manages the impact of condition on his or her physical, emotional and social life and (vi) adopts a lifestyle that promotes health”.

Several related concepts used in the context of self‐management are relevant to care provision: self‐management ability, self‐management behaviour and self‐management support. Self‐management ability is the ability to manage key internal and external personal resources,^1^ and self‐management behaviour can be viewed as the application of self‐management abilities. Self‐management support concerns the care and education offered to improve a person's self‐management skills: this is needed when improving and maintaining self‐management ability and behaviour. Reliable and valid measurement instruments are needed to keep track of these self‐management abilities, behaviour and need for support of older adults. Such measurement instruments are not yet available for the self‐management behaviour of community‐living older adults, although they are for self‐management abilities^8^ and perceived self‐management support.^9^ A measurement instrument for self‐management behaviour is important to assess the status and to follow the development of someone's self‐management behaviour over time. This insight gives professionals and older adults the opportunity to tailor the level of care and support to the individual needs of older adults.

A likely candidate for measuring self‐management behaviour in community‐living older adults is the Partners in Health (PIH) scale, developed to assess the self‐management knowledge and behaviour in people with chronic conditions.^6^ The PIH scale was developed by Battersby et al.^6^ and is based on the aforementioned self‐management principles. It has been validated for Australian and Mexican patients with chronic conditions, such as diabetes, cardiovascular disease and hypertension, and has been translated into Spanish.^7^, ^10^ Although the PIH scale aims to assess the self‐management knowledge and behaviour in patients with chronic conditions,^6^, ^7^ it has the potential to measure the self‐management knowledge and behaviour in a population of community‐living older adults. Therefore, the aims of this study were to design a measurement instrument, that is the Partners in Health scale for older adults (PIH‐OA), to assess the self‐management knowledge and behaviour of healthy and unhealthy community‐living older adults and to examine its psychometric properties in a Dutch context.

## 2 Methods

### 2.1 Study design and sample

We used the cross‐sectional data from the second measurement wave (i.e. 12 months of follow‐up after baseline) of a postal survey (N=1127, 77% of baseline population, mean age 81.7, SD=4.5), which was conducted within the framework of the Embrace study (SamenOud in Dutch). The Embrace study is a stratified randomized controlled trial among Dutch participants, which started in January 2012 and which aims to examine the effectiveness of an integrated elderly care model for community‐living older adults. A more detailed description of the design, samples and procedures can be found elsewhere.^11^ The Medical Ethics Committee of the University Medical Center Groningen assessed the study protocol and concluded that approval was not required (Reference METc2011.108). All the participants had provided informed consent.

### 2.2 Measures

Self‐management knowledge and behaviour was measured using the PIH‐OA, the adapted version of the PIH scale. The original PIH scale consists of twelve items from four subscales: “Knowledge”, “Coping”, “Management of symptoms” and “Adherence to treatment”.^7^ Response options range from zero to eight, with higher scores indicating better self‐management knowledge and behaviour.

The PIH‐OA was adapted to the context of community‐living older adults in five steps. First, the PIH‐OA was translated into Dutch by three Dutch researchers with the operational competence in English at university level. Differences were resolved by discussion. Second, to make the PIH‐OA suitable for all older adults, either healthy or unhealthy, the term “health condition” from the original version was replaced by “consequences of ageing”. Third, items two and three of the original version were split into two new items. Item two “Overall, what I know about the treatment, including medications of my health condition(s) is…” was replaced by “In general, what I know about care and support for the consequences of growing older is…” and “In general, what I know about the medication I am taking is…”. Item three “I take medications or carry out the treatments asked by my doctor/health worker” was replaced by “I take my medication in accordance with the prescription” and “I perform activities to remain healthy or improve my health as agreed”. Fourth, the preliminary Dutch version of the PIH‐OA was pretested for clarity, comprehensiveness, redundancy and participant burden in a random sample of eight community‐living older adults (five women and three men aged 61–84). This pretest showed that no further modifications were needed. Fifth, the preliminary Dutch version of the PIH‐OA was retranslated back into English by two native speakers.

The five steps resulted in a preliminary Dutch and English version of the PIH‐OA with 14 items. Scoring options ranged from zero (a little/sometimes) to eight (a lot/always), with higher scores indicating the better self‐management knowledge and behaviour. To prevent missing values, the response option “not applicable” was introduced for care‐related items (items three to eight), as they were not relevant for healthy older adults.

Health Status was measured using the EuroQol‐5D three‐level version (EQ‐5D‐3L) and the EuroQol‐5D Visual Analogue Scale (EQ‐5D‐VAS).^12^ The EQ‐5D‐3L measures five dimensions of health, namely the following: “Mobility”, “Self‐care”, “Usual activities”, “Pain/discomfort” and “Anxiety/Depression”. Each dimension is measured with one item and three response options: no problems, some problems and extreme problems. A total index score was calculated, and higher scores indicated better health status.^13^ For the EuroQol‐5D Visual Analogue Scale (EQ‐5D‐VAS), response options ranged from 0 (worst imaginable health state) to 100 (best imaginable health state).

Perceived integrated care was measured using the Dutch version of the Patient Assessment of Integrated Elderly Care (PAIEC).^9^ The subscale “Patient activation and contextual information” contained seven items, the subscale “Goal setting and problem‐solving” seven items and the subscale “Coordination and follow‐up” six items. Response options range from zero to five, with higher scores indicating a better quality of integrated care perceived by community‐living older adults. The internal consistency of the PAIEC scales and subscales was good (α ranging from .91 to .97).

Activities of daily living (ADL) were measured using the modified Katz ADL index.^14^ The Katz ADL index measures eight physical and seven instrumental ADL. Response options are zero (Yes, I am able to perform this activity) and one (No, I am not able to perform this activity). Higher scores indicate worse functional status. The internal consistency of the Katz ADL index was good (KR‐20=.84).

### 2.3 Data analyses

Sample characteristics were analysed using descriptive statistics.

Acceptability was determined by calculating the missing data per item. According to Hobart et al.,^15^ a scale score can be calculated when more than 50% of the items are completed. The response option “not applicable” was recorded into missing. Missing items were replaced by the mean score based on the number of items and the ordinal alpha, after defining the factor structure.^16^

#### 2.3.1 Factor structure

Explanatory factor analysis (EFA) was performed to explore the factor structure. Items were treated as ordinal variables with a robust weighted least‐square method estimator and using oblique rotation.^17^ To assess the goodness of fit of the model, the following fit measures were considered (with ideal cut‐off values indicated): root‐mean‐square error of approximation (RMSEA ≤.06), standardized root‐mean‐square residual (SRMR ≤.08), comparative fit index (CFI ≥.95) and Tucker–Lewis index (TLI≥.95).^18^ Items with a factor loading of >.40 on their own factor and <.3 on other factors were assigned to the potential factor.

#### 2.3.2 Internal consistency

Ordinal Cronbach's alpha coefficients were calculated for the total score and for each subscale. An ordinal Cronbach's alpha of .70 or higher is regarded as good.^19^

#### 2.3.3 Known groups validity

Known groups validity was examined using a Mann–Whitney test. No statistically significant differences were expected between males and females, age groups (two groups: <82 and ≥82) and marital status (two groups: married or in a long‐term relationship and widowed, divorced or single). It was hypothesized that participants with medium or high education levels would have the better self‐management knowledge and behaviour scores than participants with low education levels. Furthermore, it was hypothesized that participants with good health status (scores ≥60) would have better self‐management knowledge and behaviour scores than participants with poor health status (score <60). The effect sizes for nonparametric tests were calculated to reveal clinically relevant differences (*r*≥.10).^20^, ^21^

#### 2.3.4 Discriminant validity

To examine whether the PIH‐OA total scale and subscales measure other constructs such as health status, perceived integrated care and ADL, correlations between the PIH‐OA total scale and subscales and EQ‐5D‐3L, EQ‐5D‐VAS, the PAIEC total scale and subscales and the Katz ADL index were calculated using Spearman's rank correlations (*r*<.3 = weak, *r* .3–.70 = moderate, *r*>7 = strong^21^). Discriminant validity was supported by weak correlations between constructs. Weak correlations were expected between the PIH‐OA total scale and subscales and the total scale and subscales of the PAIEC and Katz ADL index.

The EFA was performed using Mplus version 7.1 (Muthén & Muthén, Los Angeles, CA, USA). All other analyses were performed using SPSS version 23 (IBM SPSS Statistics for Windows, Version 23.0. IBM Corp., Armonk, NY, USA).

## 3 Results

### 3.1 Sample characteristics

A total of 1127 participants completed the PIH‐OA and were included in the analyses. The mean age of the participants was 81.7 (SD=4.48, range 75–100). Table 1 shows the sample characteristics.

**Table 1:** Sample characteristics and known groups validity of the Partners in Health scale for older adults (PIH‐OA) (N=1127)

	N (%)
Gender	
Male	501 (44.5)
Female	626 (55.5)
Age	
<82 years of age	688 (61.0)
≥82 years of age	439 (39.0)
Marital status	
Married or in a long‐term relationship	644 (58.1)
Divorced, widowed or single	464 (41.9)
Education level	
Low	564 (50.5)
Medium/high	554 (49.5)
Health status (EQ‐5D‐VAS)	
Poor	197 (17.5)
Good	930 (82.5)

### 3.2 Acceptability

A high percentage of participants (ranging from 11.6% to 62.2%) responded “not applicable” to the six items on receiving care (items three to eight), which represents poor acceptability.^15^ Therefore, we analysed the six deleted items further, as it might be that these items were only valid for unhealthy community‐living participants. To test this assumption, we selected a subsample of participants from the intervention group who were frail or had complex care needs (and left participants without care needs out of consideration). Selecting participants from the intervention group ensured us that the selected participants had received care (91.9% received care and 72.2% used more than four medicines). Still, a substantial portion of the subsample (3.6% to 46.4%) responded “not applicable”, indicating that the six deleted items were not valid for both healthy and unhealthy participants. Based on these findings, the six items on receiving care were deleted from further analysis. Table 2 shows the deleted items.

**Table 2:** Items from the preliminary Partners in Health scale for older adults (PIH‐OA) and results of the explanatory factor analysis (N=1127)

Items		Factor 1	Factor 2	Factor 3
1.	In general, this is what I know about the consequences of growing older	**.915**	−.009	−.017
2.	In general, this is what I know about care and support for the consequences of growing older	**.816**	.027	.024
9.	I keep an eye on the consequences of growing older and the signals my body sends me (such as walking with increased difficulty, less contact with others, more difficulty with housework and less fitness)	−.022	**.967**	−.011
10.	I take action when my body sends me signals that I am not very well, or when I notice that the consequences of growing older are becoming more serious for me	.036	**.656**	.130
11.	I am able to deal with the consequences of growing older in relation to my physical activities (for example walking or doing housework)	−.006	.036	**.818**
12.	I am able to deal with the consequences of growing older in relation to my feelings (such as emotions and spiritual wellbeing)	.071	−.010	**.878**
13.	I am able to deal with the consequences of growing older in relation to my social life (for example contact with other people)	.003	−.012	**.836**
14.	In general, I am able to live healthily (for example not smoking, moderate alcohol consumption, healthy eating or regular exercise)	−.100	.163	**.462**
Deleted items				
3.	In general, this is what I know about the medication I am taking			
4.	I take my medication in accordance with the prescription			
5.	I perform activities to remain healthy or improve my health as agreed			
6.	I decide about care and supervision along with the relevant care provider			
7.	I am able to arrange care and support with my care provider which takes account of what I think is important			
8.	I go to appointments arranged for me by my care provider			

Note: The bold regressions coefficients indicate on which factor the item predominantly loaded.

### 3.3 Factor structure and scale construction

The results of the EFA, based on eight items, are presented in Table 2. Factor loadings pointed to a three‐factor model. The fit measures for the goodness of fit of the model were good: RMSEA=.04, SRMR=.01, CFI=.99, TLI=.99. The subscales were defined as follows: Knowledge (items one and two), Management (items nine and 10) and Coping (items 11–14).

### 3.4 Internal consistency

For the total scale, if any item was missing, it was replaced by the mean score of the items of that scale. Ordinal alphas were calculated for the total scale and for each subscale to examine the internal consistency. All scales showed good internal consistencies (α ranging from .77 to .84, see Table 3).

**Table 3:** Descriptive information on the Partners in Health scale for older adults (PIH‐OA) total scale and its subscales (N=1127)

	Items (K)	Cases (N)	Possible scale scores	Observed scale scores	% Lowest score	% Highest score	Ordinal alpha
Total	8	1118	8–64	9–64	0.09	1.25	.77
Knowledge	2	1114	2–16	2–16	5.30	6.28	.84
Management	2	1112	2–16	2–16	2.61	24.19	.77
Coping	4	1107	4–32	4–32	0.18	8.76	.83

### 3.5 Known groups validity

Table 4 shows the results of the known groups validity tests. The results show that the PIH‐OA total scale and subscales did not discriminate between gender, age groups and groups based on marital status. The participants with higher education levels or good health status reported better self‐management knowledge and behaviour than participants with low education levels or poor health status. The effect sizes calculated showed trivial‐to‐moderate clinically relevant differences between the education level and health status subgroups.

**Table 4:** Results of the known groups validity test of the Partners in Health scale for older adults (PIH‐OA) total scale and subscales (N=1127): median scores (inter‐quartile ranges)

	PIH‐OA total scale	PIH‐OA subscales		
		Knowledge	Management	Coping
Gender				
Male	50.0 (42.0–55.0)	12.0 (9.0–14.0)	14.0 (11.0–15.0)	26.0 (22.0–29.0)
Female	50.0 (42.0–55.0)	12.0 (9.0–14.0)	14.0 (11.0–16.0)	26.0 (22.0–29.0)
Age				
<82 years of age	50.0 (42.0–55.0)	12.0 (8.0–14.0)	14.0 (11.0–15.0)	26.0 (22.0–29.0)
≥82 years of age	50.0 (43.0–55.0)	12.0 (9.0–14.0)	14.0 (11.0–15.0)	25.0 (22.0–28.0)^a^
Marital status				
Married or in long‐term relationship	50.0 (42.0–55.0)	12.0 (9.0–13.0)	14.0 (11.0–15.0)	26.0 (22.0–29.0)
Divorced, widowed or single	50.0 (43.0–55.0)	12.0 (9.0–14.0)	14.0 (11.0–16.0)	25.0 (22.0–29.0)
Educational level				
Low	48.0 (41.0–54.0)	11.0 (8.0–13.0)	13.0 (10.0–16.0)	25.0 (21.0–28.0)
Medium/High	52.0 (44.6–56.0)^b^	12.0 (10.0–14.0)^b^	14.0 (12.0–15.0)^a^	26.0 (23.0–29.0)^b^
Health status (EQ‐5D‐VAS)				
Poor	43.0 (37.0–51.0)	11.0 (8.0–13.0)	13.0 (10.0–15.0)	21.0 (17.0–25.0)
Good	51.0 (44.0–56.0)^b^	12.0 (9.0–14.0)^a^	14.0 (11.0–16.0)^a^	26.5 (23.0–29.0)^c^

Effect sizes of PIH subscale score differences between the relevant characteristic categories: ^a^Trivial (*r*<.10); ^b^Small (*r*≥.10 to <.30); ^c^Moderate (*r*≥.30 to <.50).

### 3.6 Discriminant validity

The correlations between almost all of the PIH‐OA scales and the EQ‐5D‐VAS, PAIEC and Katz ADL index were weak (*r*<.30) (Table 5). An unexpected moderate correlation between the PIH‐OA “Coping” subscale, the EQ‐5D‐3L, EQ‐5D‐VAS and the Katz ADL index was found (*r*=.46 and −.41, respectively).

**Table 5:** Discriminant validity of the Partners in Health scale for older adults (PIH‐OA) total scale and its subscales (N=1127)

	Median (Interquartile Range)	Alpha/KR‐20^a^	Total PIH‐OA scale	PIH‐OA subscales		
				Knowledge	Management	Coping
Health status						
EQ‐5D‐VAS	75.0 (60.0–80.0)	x	.29**	.06*	.11**	.46**
EQ‐5D‐3L	0.81 (0.69–0.89)	.82	.25***	.02	.07*	.43***
Perceived integrated care (PAIEC)	20.0 (20.0–29.0)	.94	−.06*	.12**	−.02	−.22**
Patient activation and contextual information	7.0 (7.0–11.0)	.87	−.06	.11**	−.03	−.20**
Goal setting and problem‐solving	7.0 (7.0–9.0)	.89	−.06*	.09**	−.03	−.19**
Coordination and follow‐up	6.0 (6.0–8.0)	.84	−.06*	.08**	−.02	−.20**
Activities of daily living (Katz ADL index)	0.0 (1.0–3.0)	.84	−.25**	.01	−.10**	−.41**

x: Health status was measured using a single item, and scale reliability could not be calculated.

Spearman's rank order correlations (0.00–0.29 weak; 0.30–0.69 moderate; 0.70–1.00 strong).

^a^KR‐20 was calculated for dichotomous variables, Cronbach's alpha for all other variables.

* *P*<.05; ** *P*<.01; *** *P*<.001.

## 4 Discussion

The original PIH scale was adapted in this study for the context of community‐living older adults and translated into Dutch. This yielded an eight‐item PIH‐OA assessing the self‐management knowledge and behaviour with regard to the consequences of ageing of older adults, either healthy or unhealthy. A sufficient three‐factor structure was found with three subscales, labelled as “Knowledge”, “Management” and “Coping”. The PIH‐OA scale and its subscales showed reasonable construct validity and good internal consistency.

To achieve an acceptable fit for the target population, we had to delete six items from the preliminary PIH‐OA, that is the items on receiving care. By the deletion of the six care‐related items, the focus of the PIH‐OA has moved to self‐management behaviour regarding the consequences of ageing, instead of care as the original PIH scale did. As a result, the PIH‐OA can be used for all older adults, either healthy or unhealthy.

Adding the “not‐applicable” response option revealed how frequently it was used. Several explanations are possible. First, the PIH‐OA might not be suitable for the population of frail participants or participants with complex care needs. However, our analyses of the subsample with these participants showed that this explanation is not very likely, as almost all participants received treatment and, despite this, used the “not applicable” response option very often. Second, it is possible that the questions were too difficult, as 50% of the participants had a low education level. Third, another possible explanation is that the question content was invalid and/or that there is a cultural mismatch in their formulation. Further research among different subsamples is needed to examine the validity of these six items. The question remains whether the answers provided in the prior PIH scale validation studies are valid, that is would participants in these studies have also used the “not applicable” response option if it had been available? It may be of interest to re‐examine the content validity of the PIH items after adding the “not applicable” response option among chronic patient samples.

Our hypotheses on known groups validity and discriminant validity were mostly confirmed, that is we found lower scores on the “Coping” subscale among older participants (i.e. >82 years of age) and moderate correlations between the “Coping” subscale and “Health status” (EQ‐5D‐3L and EQ‐5D‐VAS) and “ADL” (Katz ADL index). These results suggest that coping with the consequences of getting older becomes harder for older adults. It is known that losses (e.g. relatives, friends, social roles) increase due to the ageing process.^22^ Furthermore, older adults have to face increasing dependency and loss of control.^3^ It is possible that such increases in loss and dependence result in decreased motivation among older adults to deal with the consequences of ageing. This decrease in motivation could result in a loss of health and functional status (as measured using the EQ‐5D‐3L, EQ‐5D‐VAS and Katz ADL). The loss of health and functional status could also lead to decreased motivation, resulting in a downward spiral.

This study's strengths are its large sample size and inclusion of a population fully representative of older adults in the community, that is a mix of healthy and unhealthy older adults. Another strength is the use of the COSMIN checklist as a guide.^23^ Furthermore, we used robust nonparametric tests to assess the psychometric properties of the PIH‐OA.

Using a questionnaire to measure the self‐management among older adults is a possible limitation. Participants may have provided socially desirable answers and presented themselves as better self‐managers than they really are. Future research is required to explore whether other methods offer better insights into the self‐management behaviour in older adults (e.g. via webcam or tablet or by multiple informants). Another possible limitation is that we were unable to assess the criterion validity of the PIH‐OA by comparing the results of the PIH‐OA scale with the results of a gold standard. Unfortunately, such a gold standard is not available.

For practice, the availability of a reliable and valid measurement instrument of self‐management behaviour implies that we can evaluate the extent of self‐management behaviour in all older adults. Keeping track of the self‐management behaviour of older adults could help professionals provide tailored support to improve the well‐being and independence of older adults. The validation of the PIH‐OA scale provides future research the opportunity to explore the relationship between self‐management concepts, that is self‐management ability, self‐management behaviour and self‐management support. Further examination of the six deleted care‐related items is needed to improve our understanding of self‐management behaviour regarding received care. To improve the quality of self‐management support, this information is likely to be very valuable. In addition, future research is required to examine the test–retest reliability and responsiveness of the PIH‐OA scale.

In sum, designing and validating the PIH‐OA for healthy and unhealthy community‐living older adults were of great importance to measuring and evaluating the self‐management behaviour and knowledge in this population. Although the original PIH scale was developed for patients with a chronic health condition, the instrument proved suitable for community‐living older adults. This study's results suggest that the PIH‐OA scale is thus suitable for all community‐living older adults, regardless of their health status.

## Funding Sources

This study was funded by the Netherlands Organization for Health Research and Development (ZonMw) file number 314010201 and the Dutch Healthcare Authority (NZA) file number 300‐1021. The Netherlands National Trial Register: NTR3039.

## Conflict of Interest

All authors report no biomedical financial interests or potential conflict of interests.

## References

[1] Steverink N , Lindenberg S , Slaets JPJ . How to understand and improve older people's self‐management of wellbeing. Eur J Ageing. 2005;2:235–244. 10.1007/s10433-005-0012-y PMC5546286 28794738

[2] Kennedy A , Bower P , Reeves D , et al. Implementation of self management support for long term conditions in routine primary care settings: cluster randomised controlled trial. Br Med J. 2013;346:f2882. 23670660 10.1136/bmj.f2882 PMC3652644

[3] Spoorenberg SLW , Wynia K , Fokkens AS , Slotman K , Kremer HPH , Reijneveld SA . Experiences of community‐living older adults receiving integrated care based on the Chronic Care Model: a qualitative study. PLoS One. 2015;10:e0137803. 26489096 10.1371/journal.pone.0137803 PMC4619446

[4] Rask KJ , Ziemer DC , Kohler SA , Hawley JN , Arinde FJ , Barnes CS . Patient activation is associated with healthy behaviors and ease in managing diabetes in an indigent population. Diabetes Educ. 2009;35:622–630. 19419972 10.1177/0145721709335004

[5] Lorig K . Self‐management of chronic illness: a model for the future (self care and older adults). Generations. 1993;17:11–14.

[6] Battersby MW , Ask A , Reece MM , Markwick MJ , Collins JP . The Partners in Health scale: the development and psychometric properties of a generic assessment scale for chronic condition self‐management. Aust J Prim Health. 2003;9:41–52.

[7] Petkov J , Harvey P , Battersby MW . The internal consistency and construct validity of the partners in health scale: validation of a patient rated chronic condition self‐management measure. Qual Life Res. 2010;19:1079–1085. 20437206 10.1007/s11136-010-9661-1

[8] Schuurmans H , Steverink N , Frieswijk N , Buunk BP , Slaets JPJ , Lindenberg S . How to measure self‐management abilities in older people by self‐report. The development of the SMAS‐30. Qual Life Res. 2005;14:2215–2228. 16328901 10.1007/s11136-005-8166-9

[9] Uittenbroek RJ , Reijneveld SA , Stewart RE , Spoorenberg SLW , Kremer HPH , Wynia K . Development and psychometric evaluation of a measure to evaluate the quality of integrated care: the Patient Assessment of Integrated Elderly Care. Health Expect. 2016;19:962–972. 26230633 10.1111/hex.12391 PMC5042070

[10] Peñarrieta‐de Córdova I , Barrios FF , Gutierrez‐Gomes T , Piñonez‐Martinez MDS , Quintero‐Valle LM , Castañeda‐Hidalgo H . Self‐management in chronic conditions: partners in health scale instrument validation. J Nurs Manag. 2014;20:32–37. 10.7748/nm2014.02.20.10.32.e1084 24571163

[11] Spoorenberg SLW , Uittenbroek RJ , Middel B , Kremer BPH , Reijneveld SA , Wynia K . Embrace, a model for integrated elderly care: study protocol of a randomized controlled trial on the effectiveness regarding patient outcomes, service use, costs, and quality of care. BMC Geriatr. 2013;13:62–73. 23782932 10.1186/1471-2318-13-62 PMC3702391

[12] Herdman M , Gudex C , Lloyd A , et al. Development and preliminary testing of the new five‐level version of EQ‐5D (EQ‐5D‐5L). Qual Life Res. 2011;20:1727–1736. 21479777 10.1007/s11136-011-9903-x PMC3220807

[13] Lamers LM , Stalmeier PFM , McDonnell J , Krabbe PFM , Busschbach JJ . Kwaliteit van leven meten in economische evaluaties: het Nederlands EQ‐5D‐tarief. (Quality of life in economic evaluations: the Dutch EQ‐5D). Ned Tijdschr Geneeskd. 2005;149:1574–1578. 16038162

[14] Laan W , Zuithoff NPA , Drubbel I , et al. Validity and reliability of the Katz‐15 scale to measure unfavorable health outcomes in community‐dwelling older people. J Nutr Health Aging. 2015;18:848–854. 10.1007/s12603-014-0558-5 PMC12878471 25389963

[15] Hobart J , Riazi A , Lamping D , Fitzpatrick R , Thompson A . Improving the evaluation of therapeutic interventions in multiple sclerosis: development of a patient‐based measure of outcome. Health Technol Assess. 2004;8:1–60. 10.3310/hta8090 14982653

[16] Van Sonderen E . Omgaan met ontbrekende gegevens in het bijzonder bij schaal items (How to handle missing data in particular scale items). Verpleegkunde, Ned Wetenschappelijk Tijdschr voor verpleegkundigen. 2000;15:104–111.

[17] Browne MW . An overview of analytic rotation in exploratory factor analysis. Multivar Behav Res. 2001;36:111–150.

[18] West SG , Taylor AB , Wu W . Model Fit and Model Selection in Structural Equation Modeling. New York: Guilford Press; 2012.

[19] Gadermann AM , Guhn M , Zumbo BD , Columbia B . Estimating ordinal reliability for Likert‐type and ordinal item response data: a conceptual, empirical, and practical guide. Pract Assess Res Eval. 2012;17:1–13.

[20] Ivarsson A , Andersen MB , Johnson U , Lindwall M . To adjust or not adjust: nonparametric effect sizes, confidence intervals, and real‐world meaning. Psychol Sport Exerc. 2013;14:97–102.

[21] Cohen J . Statistical Power Analysis for the Behavioral Sciences. Hillsdale, NJ: Erlbaum Associates; 1988.

[22] Thumala Dockendorff DC . Healthy ways of coping with losses related to the aging process. Educ Gerontol. 2013;40:363–384.

[23] Terwee CB , Mokkink LB , Knol DL , Ostelo RWJG , Bouter LM , De Vet HCW . Rating the methodological quality in systematic reviews of studies on measurement properties: a scoring system for the COSMIN checklist. Qual Life Res. 2011;21:651–657. 21732199 10.1007/s11136-011-9960-1 PMC3323819

